# Reduction of PVA Aerogel Flammability by Incorporation of an Alkaline Catalyst

**DOI:** 10.3390/gels7020057

**Published:** 2021-05-08

**Authors:** Zhi-Han Cheng, Mo-Lin Guo, Xiao-Yi Chen, Ting Wang, Yu-Zhong Wang, David A. Schiraldi

**Affiliations:** 1 Department of Macromolecular Science & Engineering, Case Western Reserve University, Cleveland, OH 44106, USA; zxc357@case.edu (Z.-H.C.); mxg501@case.edu (M.-L.G.); xxc293@case.edu (X.-Y.C.); 2 College of Chemistry, Sichuan University, Chengdu 610064, China; wangting1993@mail.xhu.edu.cn (T.W.); yzwang@scu.edu.cn (Y.-Z.W.)

**Keywords:** aerogel, flammability, base, alkali, char

## Abstract

Sodium hydroxide was used as a base catalyst to reduce the flammability of poly(vinyl alcohol) (PVA) aerogels. The base-modified aerogels exhibited significantly enhanced compressive moduli, likely resulting in decreased gallery spacing and increased numbers of “struts” in their structures. The onset of decomposition temperature decreased for the PVA aerogels in the presence of the base, which appears to hinder the polymer pyrolysis process, leading instead to the facile formation of dense char. Cone calorimetry testing showed a dramatic decrease in heat release when the base was added. The results indicate that an unexpected base-catalyzed dehydration occurs at fire temperatures, which is the opposite of the chemistry normally observed under typical synthesis conditions.

## 1. Introduction

Aerogels are one of the lowest density families of known materials, first reported by Kistler in 1931 [1], who used silicon alkoxides as sol-gel precursors to silica, which were then carefully dried to avoid capillary collapse. The name “aerogel” means a wet gel whose solvent has been exchanged with air; these materials possess the unique properties of low densities (ranging from 0.005 to 0.1 g/cm^3^), high porosities, high specific surface areas and low thermal conductivities [2,3,4,5]. As these material properties are valuable in designing consumer and industrial products, there has been a steady increase in the use of aerogels in thermal insulation, liquid absorbents, energy storage, and catalysis [6,7,8,9,10]. Inorganic aerogels tend to be brittle, which has led to the evaluation of polymer-based aerogels, which are materials that can exhibit polymer foam-like properties [11,12]. Poly(vinyl alcohol) (PVA) is an ideal candidate for fabricating such polymer aerogels because of its good chemical stability, low toxicity and favorable mechanical properties [13]. As a water soluble polymer with abundant hydroxyl groups, PVA aerogels can be prepared using an environmentally friendly, freeze drying method [14,15,16,17].

PVA aerogels are potentially promising candidates for the replacement of traditional polymer foams in insulation, packaging and building areas; these polymer foams suffer from their inherent flammability [3,17]. More than 1.3 million fires were reported in 2017 in the United States alone, resulting in an estimated 3400 civilian deaths and $23 billion in property loss, hence the importance of materials flammability cannot be over emphasized [18]. Flame retardants can be incorporated into polymer systems, but often suffer from toxicity and/or mechanical properties issues; additives such as polybrominated diphenyl ethers (PBDEs) and polybrominated biphenyls (PBBs) are often effective in limiting polymer flammabilities, but are under increasing scrutiny for toxicological reasons [19,20,21]. The few known flame retardants that could potentially resolve the toxicity/mechanical properties problems require high loadings, increasing final product densities and costs [22], limiting their commercial attractiveness [23,24]. The thermal degradation of PVA has been addressed by some authors, and to a limited extent the flammability of this polymer has also been explored [25,26,27,28]. These previous works have focused on dense films and molded PVA samples; one of those studies also showed that added NaOH could lower the flammability of the system, though no explanation for the effect was given [25].

In the present work, we report a novel method of increasing the mechanical properties of PVA aerogels, while decreasing their flammabilities, maintaining low densities, and making use of a low cost/toxicity additive. Sodium hydroxide (NaOH) can act as a catalyst at flame temperatures to promote a char forming process without the addition of other flame retardants. Low additive levels of NaOH were found to profoundly alter the aerogel properties, minimizing their impact on product density. To the best of our knowledge, there is no similar system of PVA aerogels that incorporates low levels of NaOH, nor of a systematic study varying that additive’s concentration previously reported in the literature.

## 2. Results and Discussion

The aerogel compositions examined in this study are given in Table 1.

### 2.1. Apparent Density and Mechanical Performance

The apparent densities of the aerogels are shown in Table 2. No significant differences in densities were noted, which is consistent with the percentage of solids used for each of the compositions; the exception to this observation occurred in P_5_/S_0.5_ when a large amount of sodium hydroxide was added to the formulation, increasing the aerogel density. No obvious shrinkage was observed after freeze-drying and all samples maintained good shape in the mold during the process. While the mechanical properties of the PVA aerogel were reported to decrease with the addition of inorganic matter (because the poor interfacial adhesion between them) [29], the addition of NaOH actually increased the mechanical properties of PVA aerogels produced in this study. The initial compressive modulus of P5/S0.5 was 1.30 ± 0.30 MPa, which is nearly four times that of the control, potentially because phase separated NaOH could densify the solid “struts” in the aerogel structure

To eliminate the influence of density, the specific compressive modulus was also compared. The specific moduli were calculated by dividing the ultimate compressive modulus values by the sample densities. As can be seen in Table 2, the specific compressive moduli increased from 4.8 ± 1.6 to 16.3 ± 3.0 MPa cm^3^/g as the NaOH was increased from 0% to 9.1%. The increases in mechanical properties of the aerogels with increasing NaOH levels differs from the observed mechanical properties of PVA films, which incorporate that same additive [28]. Our previous work with PVA aerogels has shown that their mechanical properties are extremely sensitive to the skeletal density of aerogel “struts”, which resemble the cellular walls in foams, as well as the morphology of the aerogels [3,5,11,12,13]. It has also been demonstrated that increasing levels of hydrogen bonding within PVA aerogels can significantly enhance their mechanicals properties [3]. It is not surprising that even low levels of additives, capable of binding to the polymer via hydrogen bonding, would bring about skeletal densification and morphological changes.

### 2.2. Morphology

**Figure 1:**
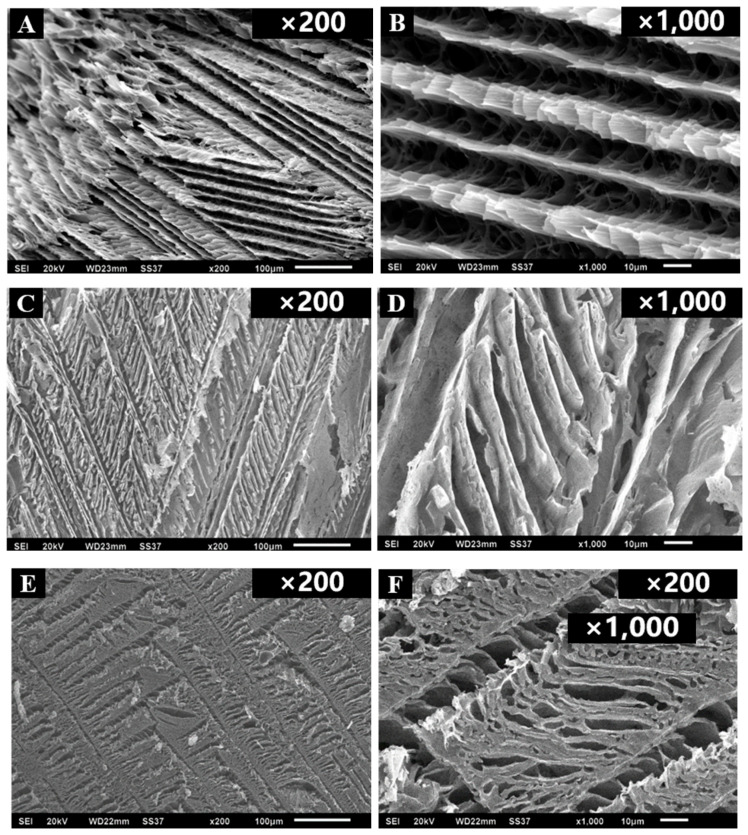
SEM images of aerogel samples. (**A**,**B**) P_5_; (**C**,**D**) P_5_/S_0.001_, and (**E**,**F**) P_5_/S_0.5_. P_x_/S_Y_ defines the polymer and sodium hydroxide concentrations used to produce the aerogels.

SEM was used to investigate the aerogel morphologies and the relationship between the structure and properties. Figure 1A,B shows typical “house of cards” aerogel structures, which is a lamellar structure caused by ice growth [30,31]. While with the addition of 0.02% NaOH, the lamellar structure was retained, the structure of the individual layers was not as neat as those of unmodified PVA aerogels samples. As the level of NaOH added to the polymer was increased, structural changes were observed—previous work has shown that mechanical properties trend with an aerogel structure [32]. In samples wherein the amount of NaOH was increased to 3.8%, the lamellar structure was interrupted (Figure 1E,F), gallery spaces decreased in size and increased in number; the samples shrank/densified, with a concomitant increase in compressive moduli (typical of such aerogels).

### 2.3. Thermal Stability & Degradation Mechanism

**Figure 2:**
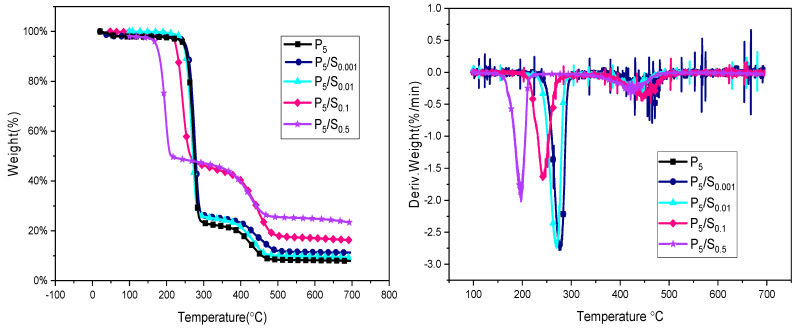
TGA and DTG (differential gravimetry) curves of PVA and PVA/NaOH aerogels at a heating rate of 10 °C/min under nitrogen.

**Figure 3:**
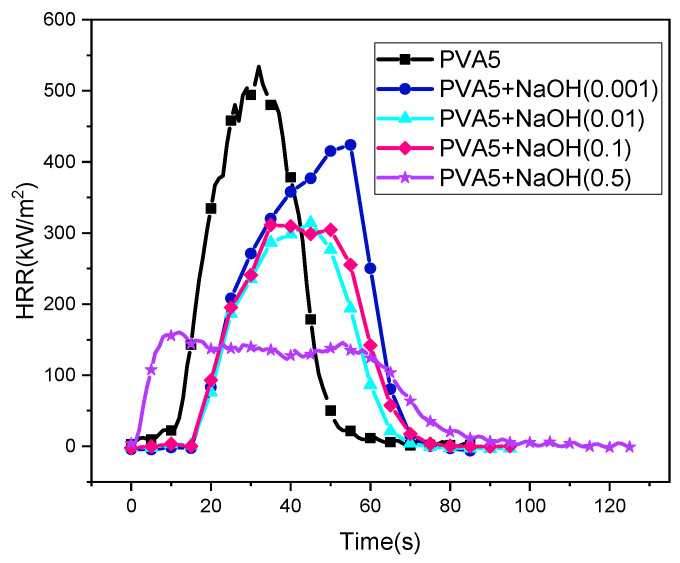
HRR plots of PVA and PVA/NaOH aerogels.

The thermal stabilities of the aerogels in this study were investigated by thermogravimetric analysis (TGA; Figure 2) and differential thermogravimetry (DTG; Figure 3). The related data are shown in Table 3, which includes the decomposition temperatures at 5% weight loss (Td_5%_), at 20% weight loss (Td_20%_), and at the maximum decomposition rates (Td_max_), the values at maximum mass decomposition rate (dW/dT) and the char. PVA aerogels could easily capture water from the atmosphere because the existence of abundant hydroxyl groups on its chain. To avoid the influence of moisture, the samples were heated to 100 °C from room temperature at a heating rate of 40 °C/min and then equilibrated at 100 °C for 2 min. After that it was heated to 700 °C at a heating rate of 10 °C/min under nitrogen.

**Scheme 1:**
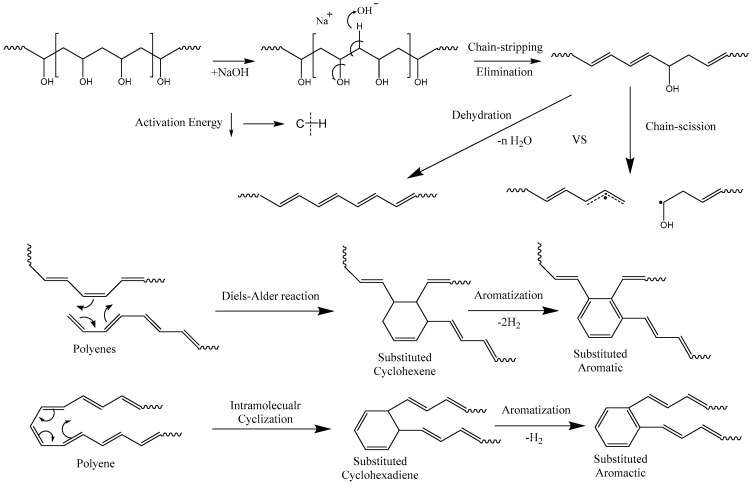
Thermal pyrolysis process of PVA with the addition of NaOH.

As hydrophilic materials rich in surface hydroxyl, PVA aerogels were difficult to remove all physisorbed water by regular drying methods. The weight loss stage before 100 °C is attributed to the loss of this water. The main decomposition step occurred between 150–500 °C. The onset decomposition temperature was evaluated by Td_5%_. With the addition of NaOH, the onset temperature decreased, which is consistent with previously reported observations [31,33]. We propose that NaOH is acting as a base catalyst at the elevated temperatures associated with burning polymers. In this mechanism, which is speculative at this time, PVA undergoes a rapid chain-stripping elimination of water once it is heated above the decomposition temperature (shown in Scheme 1) [34,35]. The chain stripping reaction (which is known chemistry in the absence of base catalysis) could produce polyenes through dehydration, which is in a competitive relationship with chain scission. Such polyenes are char precursors. With base catalysis, hydroxide may decrease the C-H bond strength, decreasing the onset decomposition temperature of the PVA in the aerogels, producing higher levels of polyenes and ultimately char was produced rather than chain scission products. Catalysis of the mechanistic steps shown in Scheme 1 are not normally associated with alkaline materials (rather with acids); the normal mechanisms of organic chemistry are not necessarily in play at the elevated temperatures associated with fire events—activation energies for reactions can be overcome when reaction temperatures are increased by hundreds of degrees. Recent innovations in high temperature alkaline catalysis (sodium hydroxide and calcium carbonate) used in biodiesel refining [36,37], as well as in the Guerbet Coupling reaction [38], suggest that the hypothetical mechanism shown in Scheme 1 has a prior art basis. In the most generally-accepted mechanism for the Guerbet Coupling of ethanol to butanol (or of butanol to 2-ethylhexanol), the high temperature dehydration of an alcohol is a key step, just as it is in the chain stripping mechanism. Hence, new chemistries may need to be considered when examining the mechanisms of flame retardation of organic polymers.

As can be seen in Table 3, the onset temperature for PVA aerogels decreased from 246 °C to 171 °C with an increasing base catalyst. A change in the degradation mechanism, enhancing chain stripping at the expense of chain scissions, was similarly observed with increasing levels of the base catalyst (Figure 2 and Figure 3). With the greater extent of chain stripping in the presence of base, more char (less weight loss) resulted. In the proposed char-forming pathway, the polyenes undergo a Diels-Alder or intramolecular cyclization reaction [34,35], producing substituted cyclohexenes and cyclohexadienes, respectively, which can then aromatize to substituted aromatics. The fusion of aromatics results in the final, observed char products [39]. With the increase in char yield from 7.9 to as much as 23.5% (at 0.5% NaOH), approximately logarithmic relations between NaOH concentration and char yield are observed, with the data point at 0.001 being the only outlier; by increasing the addition levels of the base, the maximum mass-decomposition rates (dW/dT) dropped, as expected from 2.71 to under 2.0. Previously reported work by Arora and coworkers [28], examining the effects of as much as 5 wt% NaOH to PVA films, proposes that dehydration of alcohol groups in PVA likely commence at decreased temperatures when the alkaline agent is added—this is consistent with our proposed alkaline catalysis in PVA aerogels. The previous workers observed an increase in limiting oxygen index (LOI) from 20.5 to 27.2 with the incorporation of 4.5% NaOH. These authors also suggested that water generated by alcohol dehydrations could provide a diluent effect in reducing flammability. We cannot rule out such a mechanism, but also propose the graphitization/char formation shown in Scheme 1 to play an important role in the observed flame retardation.

### 2.4. Combustion Behavior

The combustion behavior of the aerogels was studied using cone calorimetry. Cone calorimetry is widely used in fire studies, it could provide plentiful data, including the time to ignition (TTI), peak of heat release (PHRR), time to peak of heat release (TTPHRR) and total heat release (THR); the results are shown in Table 4.

The samples were ignited under the heat flux of 50 kW/m^2^. While all the samples were ignited in a short time, their overall flammability’s were significantly reduced with incorporating NaOH. Figure 3 shows the HRR plots of PVA/NaOH aerogels and the control sample. The HRR curve of pristine PVA samples exhibited a sharp peak, with a PHRR of 533 kW/m^2^; with the addition of a relatively small amount of NaOH, the curve showed a similar, relatively broad peak, and PHRR was decreased to about 310 kw/m^2^; When the amount of NaOH was “sufficient”, the HRR curve showed a very broad peaks with the lowest PHRR. The shape of P_5_/S_0.5_ aerogel was a typical HRR curve of thick charring materials [40]. The PHRR of P_5_/S_0.5_ was 160 kw/m^2^, which was 30% of the number for pure PVA aerogel. The results indicate the addition of NaOH could decrease the fire risk of PVA based aerogel material in a big fire test. No dripping was observed for any of the samples during combustion testing.

Total heat release (THR) data are listed in Table 4. THRs of aerogel samples were all lower than that of the control samples, except for P_5_/S_0.001_, which likely contained insufficient base catalyst to produce an observable effect. To eliminate the influence of mass differences and to better illustrate the combustion behavior of the samples, THR/mass was compared. The P_5_/S_0.5_ aerogel exhibited the lowest THR/mass value, which was 1.6 MJ/(m^2^ g), much lower than the results of the control PVA aerogels. The THR/mass values of P5/S0.001, P5/S0.01, and P5/S0.1 samples were similar, which is consistent with the PHRR results, indicating similar flammabilities.

**Figure 4:**
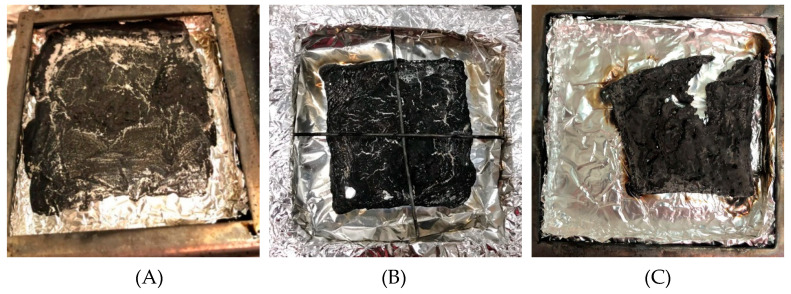
Images of aerogel samples after cone calorimetry test. (**A**) P_5_; (**B**) P_5_/S_0.01_; (**C**) P_5_/S_0.5_.

The data above all showed that the addition of NaOH decreases the flammability of the PVA aerogels. The samples containing NaOH could form char to protect the unignited matrix polymer. The samples with relatively small amounts of NaOH in the PVA system showed slightly lower flammabilities compared to the pristine PVA aerogels, but the char forming process was insufficient to protect the unignited polymer. With a sufficient level of NaOH incorporated into the aerogel system, the samples could form denser char rapidly to fully cover the unignited part. The TGA char yields, given in Table 3, support the increasing levels of char with increased NaOH addition levels (8–11% char at/under 0.01% NaOH; 16% char at 0.1% NaOH; 24% char at 0.5%); these controlled values are consistent with the qualitative observations from cone calorimetry experiments. The mechanism of graphitization of PVA under flame conditions is consistent with the literature of PVA degradation, with the largely undescribed addition of alkaline catalysis. This hampered heat transfer and isolated the unignited matrix from the ignition source. Figure 4 illustrates the chars remaining after cone calorimetry testing. The control samples burned totally with little char forming. P_5_/S_0.01_ aerogel formed slightly more char than the pristine PVA aerogel, while P_5_/S_0.5_ formed a much denser char and was sustained in a good shape, even after the test. This result is in accordance with our proposed mechanism of base catalyzed char formation.

**Figure 5:**
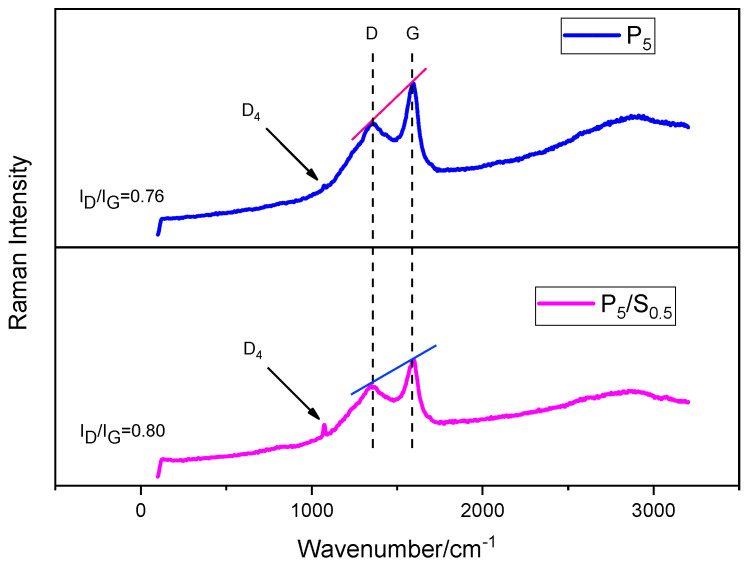
Raman spectra of char residues of P_5_ and P_5_/S_0.5._

To better understand the catalytic function of NaOH in PVA aerogel during combustion, the residues were studied using Raman spectroscopy (Figure 5). The spectra of chars with and without NaOH all exhibit two broad peaks with intensity maxima at 1580–1600 cm^−1^ (G band) and 1350 cm^−1^ (D band) [41,42]. The G band corresponds to the stretching vibration mode with E2 g symmetry in the aromatic layers of the graphite crystalline, whereas the D band is due to the disordered graphite [41,43,44]. The graphitization degree of the chars were estimated by the ratio of the intensity of the D and G bands (I_D_/I_G_) [41,45]. The I_D_/I_G_ of pristine PVA(0.76) is lower than that of the sample prepared with NaOH(0.80). While lower I_D_/I_G_ means better graphitization degree of the char and the better flame retardancy, it was more important that the graphitized carbons could realize an effective aggregate or crosslinking. The amount of char plays the key role in providing protection from the ignition source [43,45]. Both spectra exhibited a shoulder at ∼1200 cm^−1^ in the D band, assigned as D_4_, and attributed to the presence of polyene sp^2^-sp^3^ bonds or C–C and C=C stretching vibrations of polyene-like structures [46,47,48,49]. The charring residue of PVA/S had a higher D_4_ intensity than was seen with the char residue of pristine PVA aerogels, indicating more polyenes were produced during the combustion tests in the presence of the base, again consistent with the proposal that NaOH was playing the role of catalyst promoting polyenes formation. The char structure enabled by the NaOH could hinder the transfer of combustible volatiles and heat, restricting the further decomposition of the polymer matrix.

A potential concern to the addition of sodium hydroxide to the PVA aerogels prepared in this study is that this additive is both hydroscopic and water soluble. One could reasonably worry that properties could change with time and with environmental aging. To the former point, samples were allowed to age under laboratory conditions and appear not to suffer from any water uptake, perhaps due to hydrogen bonding between the polymer and additive, and environmental equilibration native to the PVA polymer itself. We have not carried out any experiments wherein the polymer is exposed to significant washing with water—this is a point that should be addressed with any polymer system that makes use of such basic catalysis.

## 3. Conclusions

PVA aerogels incorporating differing levels of NaOH were fabricated through a simple and environmentally friendly freeze-drying process. The corresponding apparent densities, compressive properties, morphologies, thermal stabilities, combustion behaviors, and Raman spectroscopy were investigated. The moduli of PVA aerogels increased with increasing NaOH concentration, likely due to higher overall levels of solids and densification of polymeric “struts” and morphological changes in the aerogels. The specific moduli increased by more than three times with the addition 9.1% NaOH. The microstructure of the aerogels showed that the gallery spaces decreased in size and increased in number with increasing base levels. While the thermal degradation onset temperature was lower with the addition of NaOH, the decomposition process was shunted to a pathway wherein higher char yields were obtained once a threshold level of the base was present. Combustion tests showed the addition of NaOH formed much higher char yields once the threshold level of NaOH was met; that char does protect the unignited polymer matrix, leading to overall lower levels of combustion. The PHRR decreased from 533 kw/m^2^ to 160 kw/m^2^ and THR/Mass decreased from 2.4 MJ/(m^2^ g) to 1.6 MJ/(m^2^ g) in the presence of the base. The results of this study suggest that NaOH can act as catalyst, promoting polyenes formation in PVA, therefore forming significant char. With a small quantity of base catalyst, the PVA aerogel is mechanically stronger and much less flammable, maintaining, the most important advantage of aerogel, low density without using any char forming agents or halogens compounds in order to decrease flammability.

## 4. Materials and Methods

Poly(vinyl alcohol) (PVA; Mw 13,000–23,000, 98% hydrolyzed, SIGMA-Aldrich, St. Louis, MO, USA) and sodium hydroxide pellets (ACS grade, >98%; Innovating Science, Avon, NY, USA) were used without further purification. Deionized (DI) water was obtained using a Barnstead RoPure low-pressure, reverse-osmosis system (Lake Balboa, CA, USA).

### 4.1. Preparation of Aerogels

The aerogel compositions are shown in Table 1. PVA solutions were prepared by stirring PVA powder with DI water for six hours at 90 °C. Sodium hydroxide was mixed with DI water under magnetic stirring at 40 °C for 30 min before addition to the PVA solutions; the solutions were mixed to the desired PVA concentrations at 50 °C until they were homogeneous suspensions (the final compositions are given in Table 1). The suspensions were then cast into a 100 mm × 100 mm × 10 mm rectangular mold (for cone calorimetry testing) or poured into 12.5 mL polystyrene vials (for compression testing) and frozen in a solid carbon dioxide/ethanol bath (−70 °C). These samples were freeze dried using a VirTis Advantage EL-85 lyophilizer, with the shelf temperature set to 25 °C, and the pressure set to under 10 μbar. The products were named as P_x_/S_y_, where P represents PVA and S represents sodium hydroxide; the subscripts indicate their content per 100 g water. P5 were prepared by same method and fabricated as the general controls. The samples were stored in a desiccator after fabrication and were dried in a vacuum oven at 50 °C for 20 min before characterization.

### 4.2. Characterization

The pH values were measured using a pH meter (Hanna Instruments, Smithfield, RI, USA). The meter was calibrated by two standard buffers (pH 4.01, 7.01) before every test; each composition was tested 3 times, with average values given in Table 1.

The apparent densities were calculated by measuring the mass and dimensions using a Mettler Toledo (Columbus, OH, USA) AB204-S analytical balance and an electronic digital caliper (RCBS); each composition was tested with 5 samples.

Compression testing was conducted on Instron model 5500 universal testing machine (Instron, Norwood, MA, USA), fitted with a 1 KN load cell and 10 mm/min compression rate. The modulus values were attained from the slope of the area under the linear portion of the stress-strain curves, using specimens measuring 20 mm in both height and diameter. Each composition was tested with a minimum of five cylindrical samples, and the results were averaged.

Thermal properties were measured by thermogravimetric analysis (TGA), using a TGA Q500 (TA Instruments, New Castle, DE, USA). The specimens were placed in a platinum pan, equilibrated at 100 °C for 3 min, then heated to 700 °C at a heating rate of 10 °C/min. The process was conducted under a nitrogen flow rate of 40 mL/min. Each composition was replicated 3 times.

The morphological microstructure of the aerogels was investigated using a JEOL JSM 5900LV scanning electron microscopy (SEM)(JEOL, Tokyo, Japan) with an accelerating voltage of 20 kV. Samples were frozen in liquid nitrogen for 20 min, then cryofractured before imaging.

The combustion behavior of the aerogels was measured using a cone calorimeter (Fire Testing Technology, East Grinstead, UK) in accordance with the ASTM E 1354 standard, with the limitation noted below. The heat flux was set to 50 kW/m^2^. The rectangular samples (100 mm × 100 mm × 10 mm) were wrapped with aluminum foil before testing. Note that smoke generation measurements were not made due to equipment limitations.

Raman spectroscopy was conducted on an inVia laser Raman spectrometer (Renishaw, Gloucestershire, UK) at room temperature with excitation provided by a 514 nm laser line.

## Figures and Tables

**Table 1:** Compositions of aerogels.

Sample	PVA (g)	NaOH (g)/(mol/L)	DI Water (g)	pH
P_5_	5	0/0	100	5.8
P_5_/S_0.001_	5	0.001/2.5 × 10^−4^	100	6.0
P_5_/S_0.01_	5	0.01/2.5 × 10^−3^	100	8.3
P_5_/S_0.1_	5	0.1/2.5 × 10^−2^	100	11.7
P_5_/S_0.5_	5	0.5/2.5 × 10^−2^	100	12.8

Poly(vinyl alcohol), PVA; sodium hydroxide, NaOH; deionized water, DI water.

**Table 2:** Observed aerogel properties.

Sample	Modulus (MPa)	Density (g/cm^3^)	Specific Modulus (MPa cm^3^/g)
P_5_	0.31 ± 0.11	0.065 ± 0.002	4.8 ± 1.6
P_5_/S_0.001_	0.36 ± 0.01	0.066 ± 0.002	5.5 ± 0.2
P_5_/S_0.01_	0.47 ± 0.08	0.067 ± 0.001	7.1 ± 1.3
P_5_/S_0.1_	0.55 ± 0.16	0.066 ± 0.002	8.3 ± 2.0
P_5_/S_0.5_	1.30 ± 0.30	0.079 ± 0.001	16.3 ± 3.0

**Table 3:** TGA (thermogravimetric analysis) data of freeze dried PVA and PVA/NaOH aerogels.

Sample	Td_5%_ (°C)	Td_20%_ (°C)	Td_max_ (°C)	Dw/dt (%/°C)	Residue (%)
P_5_	246	262	274	2.71	7.9
P_5_/S_0.001_	249	266	277	2.68	11.3
P_5_/S_0.01_	244	258	270	2.63	9.3
P_5_/S_0.1_	224	238	243	1.60	16.4
P_5_/S_0.5_	171	189	199	1.98	23.5

**Table 4:** Cone Calorimetry Data for PVA and PVA/NaOH Aerogels.

Sample	Weight (g)	TTI (s)	PHRR (kW/m^2^)	TTPHRR (s)	THR (MJ/m^2^)	THR/Mass (MJ/(m^2^ g))
P_5_	5.3	8	533 ± 35	32	12.9	2.4 ± 0.0
P_5_/S_0.001_	6.6	10	424 ± 37	55	14.0	2.1 ± 0.0
P_5_/S_0.01_	4.8	11	314 ± 13	45	9.9	2.1 ± 0.0
P_5_/S_0.1_	5.3	10	311 ± 31	35	11.2	2.1 ± 0.0
P_5_/S_0.5_	5.9	5	160 ± 20	13	9.5	1.6 ± 0.0

## Data Availability

Data is contained within the article.
